# Studying pre-treatment and ketamine-induced changes in white matter microstructure in the context of ketamine’s antidepressant effects

**DOI:** 10.1038/s41398-020-01122-8

**Published:** 2020-12-15

**Authors:** Valerie J. Sydnor, Amanda E. Lyall, Suheyla Cetin-Karayumak, Joey C. Cheung, Julia M. Felicione, Oluwaseun Akeju, Martha E. Shenton, Thilo Deckersbach, Dawn F. Ionescu, Ofer Pasternak, Cristina Cusin, Marek Kubicki

**Affiliations:** 1 Psychiatry Neuroimaging Laboratory, Brigham and Women’s Hospital, Harvard Medical School, Boston, MA USA; 2 Department of Psychiatry, Massachusetts General Hospital, Harvard Medical School, Boston, MA USA; 3 Depression Clinical and Research Program (DCRP), Department of Psychiatry, Massachusetts General Hospital, Harvard Medical School, Boston, MA USA; 4 Athinoula A. Martinos Center for Biomedical Imaging, Charlestown, Boston, MA USA; 5 Department of Anesthesia, Critical Care and Pain Medicine, Massachusetts General Hospital, Harvard Medical School, Boston, MA USA; 6 Department of Radiology, Brigham and Women’s Hospital, Harvard Medical School, Boston, MA USA; 7 VA Boston Healthcare System, Brockton Division, Brockton, MA USA

**Keywords:** Depression, Neuroscience

## Abstract

Ketamine is increasingly being used as a therapeutic for treatment-resistant depression (TRD), yet the effects of ketamine on the human brain remain largely unknown. This pilot study employed diffusion magnetic resonance imaging (dMRI) to examine relationships between ketamine treatment and white matter (WM) microstructure, with the aim of increasing the current understanding of ketamine’s neural mechanisms of action in humans. Longitudinal dMRI data were acquired from 13 individuals with TRD two hours prior to (pre-infusion), and four hours following (post-infusion), an intravenous ketamine infusion. Free-water imaging was employed to quantify cerebrospinal fluid-corrected mean fractional anisotropy (FA) in 15 WM bundles pre- and post-infusion. Analyses revealed that higher pre-infusion FA in the left cingulum bundle and the left superior longitudinal fasciculus was associated with greater depression symptom improvement 24 h post-ketamine. Moreover, four hours after intravenous administration of ketamine, FA rapidly increased in numerous WM bundles in the brain; this increase was significantly associated with 24 h symptom improvement in select bundles. Overall, the results of this preliminary study suggest that WM properties, as measured by dMRI, may have a potential impact on clinical improvement following ketamine. Ketamine administration additionally appears to be associated with rapid WM diffusivity changes, suggestive of rapid changes in WM microstructure. This study thus points to pre-treatment WM structure as a potential factor associated with ketamine’s clinical efficacy, and to post-treatment microstructural changes as a candidate neuroimaging marker of ketamine’s cellular mechanisms.

## Introduction

Major depressive disorder (MDD) is a highly prevalent psychiatric disorder that can persist throughout the lifetime and be difficult to treat. Typically, individuals with MDD are treated with monoaminergic antidepressants, which increase levels of intrasynaptic serotonin, dopamine, and/or norepinephrine. While many individuals with MDD experience symptom improvement with these medications, ~30% of patients are treatment-resistant^1^. To address the needs of individuals with treatment-resistant depression (TRD), alternative psychotropics, most notably ones that modulate the glutamatergic system, have been investigated^2^. Ketamine, a non-specific N-methyl-D-aspartate (NMDA) receptor antagonist, has been shown to be clinically effective for alleviating symptoms in TRD^3^.

Clinical trials of intravenous ketamine for TRD report that >60% of patients experience an improvement in depressive symptoms following a single infusion of ketamine, within just 24 h of treatment^4,5^. Moreover, intranasal esketamine in combination with an oral antidepressant has been approved as a treatment for adult TRD by the European Medicines Agency, Health Canada, and the U.S. Food and Drug Administration. Ketamine is thus a promising new therapeutic for TRD. Nonetheless, there are drawbacks associated with ketamine treatment: (1) ketamine produces temporary side effects, including dissociative symptoms; (2) the antidepressant effect is transient, lasting only days to a few weeks; and (3) the safety of repeated infusions and long-term use remains unclear. The development of psychotropics that mimic ketamine’s antidepressant neurobiological effects that have fewer side effects and improved long-term safety profiles is an active area of research^6^. The elucidation of ketamine’s antidepressant mechanisms of action in the brain—at both a cellular and systems level—may accelerate the development of novel antidepressants.

The current understanding of ketamine’s cellular antidepressant mechanisms comes from preclinical research. This research has shown that ketamine elicits glutamatergic activation of AMPA receptors, inducing synaptogenesis^7,8^; this synaptogenesis is necessary for ketamine-related reductions in depressive-like behaviors^7^. Accordingly, rapid modulation of synaptic function and neural connectivity are thought to contribute to ketamine’s antidepressant actions^6,8^. Moreover, changes in astrocyte activation and morphology have been implicated in ketamine’s antidepressant mechanisms^9,10^. The extent to which these preclinical findings translate to humans is not clear, however, as research investigating the effects of ketamine on the human brain has been relatively limited.

Neuroimaging is a critical tool for studying the neural effects of ketamine in humans. Given preclinical evidence that ketamine affects structural properties of neurons and glia, structural and diffusion magnetic resonance imaging (MRI) may be relevant. In two complementary structural MRI studies, Abdallah and colleagues demonstrated that smaller pre-treatment hippocampal volumes^11^, and a greater reduction in nucleus accumbens volume 24 h post-treatment^12^, were associated with a better clinical response to intravenous ketamine, suggesting that MRI may offer utility for examining the effects of ketamine in vivo. Whereas structural MRI is used to examine macroscopic structural properties of the brain (e.g., volume or cortical thickness), diffusion MRI (dMRI) is able to better characterize properties of tissue microstructure, most notably white matter (WM) microstructure^13^. Previous research has demonstrated that WM structure can be an important factor associated with clinical response to typical antidepressant medications^14,15^, yet only two studies to date have used dMRI to investigate the role of WM in patient response to ketamine treatment^16,17^.

This pilot study employed dMRI to investigate whether imaging-derived measures of pre-treatment WM microstructure, and ketamine-induced changes in microstructure, are associated with the antidepressant effects of ketamine. Specifically, we employed a two-compartment diffusion model^18^ to estimate free-water corrected fractional anisotropy (FA) in WM bundles prior to and 4 h following a ketamine infusion in patients with TRD. As this investigation is one of the earliest to apply dMRI to the study of ketamine in MDD, we sought to provide preliminary evidence regarding whether WM structure may influence ketamine’s efficacy, and to investigate if ketamine, in turn, affects WM.

## Methods

### Participants

Participants in this study were recruited through the Massachusetts General Hospital (MGH) Depression Clinical and Research Program (DCRP) between March 2016 and December 2017. Enrolled participants met the following pre-established inclusion criteria: aged 18–64 years, received a primary diagnosis of MDD via the MINI International Neuropsychiatric Interview, had a current episode ≥4 weeks in length, had a history of ≥1 failed medication trial during the current episode, were on a stable antidepressant and psychotherapy regimen for ≥28 days prior to ketamine infusion, had a treating psychiatrist who approved study participation, and were naive to ketamine treatment. Individuals were not enrolled if they met exclusion criteria due to non-MDD psychiatric conditions (past or current psychotic disorder, suicidal ideation, or homicidal ideation), substance use (a substance use disorder within the past 15 years, a lifetime history of ketamine, PCP, or LSD abuse), medication use (history of severe adverse drug reactions or medication intolerance), general health conditions (history of cardiovascular disease or abnormal electrocardiogram finding, current unstable medical illness, history of acute intermittent porphyria, history of seizures, hyperthyroidism, pulmonary disease with hypercarbia, narrow-angle glaucoma, past or current diagnosis of delirium or dementia, pregnant or breastfeeding, weight >250 pounds, left-handedness), or contraindications to receiving a MRI scan. Study procedures were approved by the MGH Institutional Review Board. All participants gave written informed consent to participate following a thorough description of study procedures. The authors affirm that all procedures contributing to this work comply with the ethical standards of the Institutional Review Board and with the Helsinki Declaration of 1975. This study was part of a Phase 4, single group assignment, open-label clinical trial (clinicaltrials.gov identifier NCT02544607), and thus did not include randomization or blinding procedures.

### Study procedures

Physical examinations, toxicology tests, electrocardiograms, and medical history assessments were conducted to ensure that participants were in good health prior to ketamine administration. Of the 25 participants assessed for eligibility for this study, 16 participants passed all screening procedures and received a ketamine infusion. The infusion consisted of a 0.5 mg/kg intravenous infusion of racemic ketamine administered at MGH over a 40 minute period by an anesthesiologist. The design of the study was as follows: participants underwent a baseline/pre-infusion MRI scan two hours prior to the start of the ketamine infusion; a study doctor administered the HAM-D28 immediately prior to the start of the infusion; participants underwent a second, post-infusion MRI scan 4 h after the start of the infusion; the study doctor administered the HAM-D28 by phone ~24 h after the ketamine infusion (with a time frame of “since the infusion”). The primary clinical outcome measure was the percent change on the HAM-D28 from baseline to 24 h post-infusion. The HAM-D28 assesses core depression symptoms including depressed mood, guilt, psychic anxiety, somatic symptoms, psychomotor retardation, and interest level in work and daily activates. Higher HAM-D28 scores indicate more severe depression.

### MRI data acquisition

Diffusion MRI data were acquired on a Siemens 3T MAGNETOM PRISMAFit at the Martinos Center for Biomedical Imaging in Charlestown, MA, USA. Diffusion images were acquired using a dual echo-spin EPI sequence (repetition time = 4000 ms, echo time = 55 ms, flip angle = 90°, acquisition matrix = 128 mm × 128 mm). Seventy axial slices were acquired with 2 mm^3^ isotropic voxels in 64 gradient directions with b = 1000 s/mm^2^. 10 b = 0 scans were additionally collected.

### MRI data processing

Diffusion scans underwent quality control procedures to ensure high data quality, including visual inspection of gradient volumes to check for severe motion artifacts and signal drop-outs. Preprocessing steps applied to the diffusion data included motion and eddy current distortion correction with FSL eddy version 5.0.6^19^, and the removal of diffusion gradients with signal drop-outs. Average in-scanner head motion was calculated for each scan using the rigid body transforms computed by eddy for each gradient volume.

Following preprocessing, free-water imaging^18^ was applied to generate free-water corrected (FW-corrected) FA scalar maps. Free-water imaging is a more advanced (compared to DTI) diffusion modeling approach that uses a bi-tensor model to estimate a free-water compartment (modeled using an isotropic tensor) and a tissue compartment (modeled using a single diffusion tensor) per image voxel. Removal of signal arising from free-water from the calculation of the diffusion tensor allows for tensor-derived measures (e.g., FA) that are more specific to biological tissue.

**Fig. 1:**
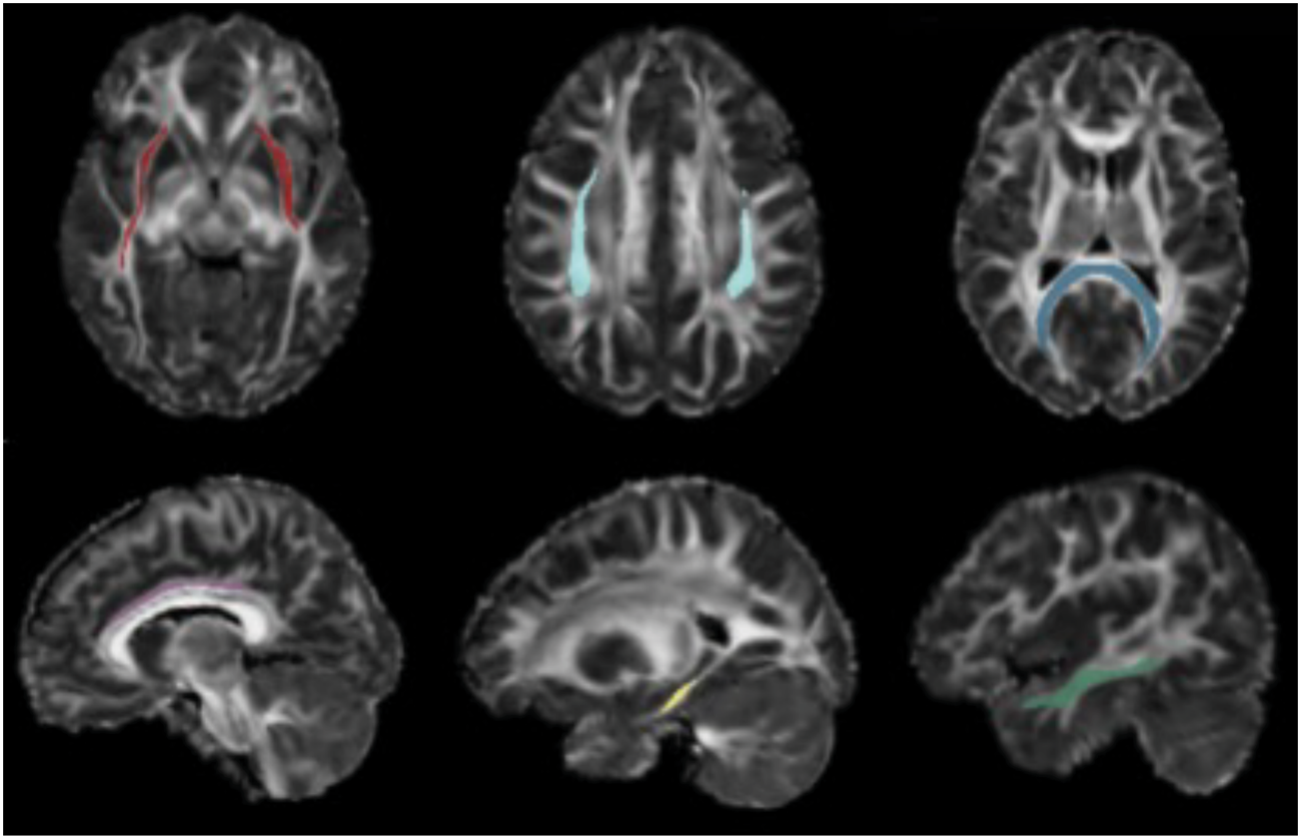
White matter bundle confidence maps. Thresholded and binarized white matter bundle confidence maps overlaid on a subject’s FA map registered to MNI space. Red: inferior fronto-occipital fasciculus; light blue: superior longitudinal fasciculus; dark blue: forceps major; purple: cingulum bundle-cingulate gyrus portion; yellow: cingulum bundle-hippocampal portion; dark green: inferior longitudinal fasciculus.

To enable quantification of mean FW-corrected FA in WM bundles in the brain, a pipeline was developed to register FW-corrected FA maps to the Illinois Institute of Technology (IIT) MNI152 mean DTI FA map^20^. For this pipeline, conventional (i.e., non-corrected) DTI FA maps were generated for all baseline and post-infusion diffusion scans and co-registered to a study group space using Advanced Normalization Tools (ANTs) multivariate template construction^21,22^. The goals of this registration to group space were to co-register pre-infusion and post-infusion scans in a manner that was unbiased to any single time-point, and to generate a study-specific DTI FA template. The study-specific DTI FA template was then non-linearly registered to the IIT MNI152 mean DTI FA map using ANTS symmetric image normalization with a cross-correlation metric. Transforms (nonlinear and affine) generated during the registration of subject DTI FA maps to group space, and during the registration of the study-specific DTI FA template to MNI152 space, were successively applied to subjects’ FW-corrected FA maps to warp them to MNI152 space. Finally, average WM bundle free-water corrected FA was estimated in the 15 major cortical bundles included in the IIT WM atlas, using major bundle confidence maps^20^ (Fig. 1). The IIT major bundle confidence maps were thresholded at 0.25 and binarized prior to measure extraction to ensure high confidence in tract localization, and to minimize gray matter-white matter partial voluming. Average pre-infusion and post-infusion FW-corrected FA were estimated for the following 15 bundles: corpus callosum (CC)-forceps major, CC-forceps minor, fornix, left and right cingulum bundle (CB)-cingulate gyrus portion, left and right CB-hippocampal portion, left and right inferior fronto-occipital fasciculus (IFOF), left and right inferior longitudinal fasciculus (ILF), left and right superior longitudinal fasciculus (SLF), left and right uncinate fasciculus (UF). The percent change in FW-corrected FA from pre-infusion to post-infusion was additionally calculated for the 15 bundles.

### Statistical analysis

Statistical analyses were conducted in R version 3.5.2. A Pearson’s correlation was used to test for an association between baseline HAM-D28 scores and percent improvement on the HAM-D28 24 h following ketamine. Partial correlations controlled for age were conducted to examine (1) associations between pre-infusion bundle FA and percent improvement on the HAM-D28 from baseline to 24 h post-infusion, and (2) associations between percent change in bundle FA from pre- to post-infusion and percent improvement on the HAM-D28 from baseline to 24 h post-infusion. Given that the size of the present patient sample is small, we utilized Cook’s distance^23^ to examine whether individual patients may have had an outsized impact on observed associations. Cook’s distance identifies influential observations (i.e., data points with large residuals and leverage) by computing how much predicted values and parameter estimates change when each observation is removed from the statistical model. We used a cut-off value of >1 for the identification of high leverage observations^24^. *Q*–*Q* plots and Shapiro–Wilk tests were used to assess residuals for any substantial deviations from normality. Paired samples *t*-tests (two-tailed) were employed to investigate within-subject change in bundle FA from pre-infusion to post-infusion. Partial correlations and paired samples *t*-tests were performed for all white matter bundles (uncorrected).

Analyses did not control for medication use as all 13 patients were on a stable psychotropic medication regimen including an antidepressant or mood stabilizer for 28 days prior to the infusion. To investigate whether in-scanner head motion impacted bundle FA, linear regressions were run with head motion and age included as predictors of FA; regressions were conducted for each WM bundle at each time-point (pre-infusion and post-infusion). A paired samples *t*-test (two-tailed) was additionally performed to examine whether participants’ average in-scanner head motion systematically differed between pre-infusion and post-infusion scans.

## Results

### Replication of ketamine’s antidepressant efficacy for TRD

Pre- and post-infusion dMRI data were available for 13 of 16 participants. Reasons for missing data (*N* = 3) included a scanner malfunction and participant requests to stop scanning prior to dMRI sequence acquisition. Demographics for the final study sample (*N* = 13) include a mean age of 42.0 years (±13.9 years), 38.5% male (5/13 participants), and 84.6% White (11/13 participants). All participants were right-handed. Clinical characteristics of this sample can be found in Table 1.

**Table 1:** Study sample clinical characteristics.

Clinical measure	Mean (SD)
Major depression age of onset	14.9 (6.5)
Number of lifetime episodes	2.8 (2.7)
Length of current episode in months	108.8 (125.1)
Number of failed medication trials (lifetime)	5.9 (4.5)
Number of failed medication trials (current episode)	4.7 (4.6)
Baseline HAM-D28 total score	24.0 (4.5)
24 h post-infusion HAM-D28 total score	8.3 (4.8)

Clinical characteristics of the 13 participants included in the final study sample.

Depression severity, as indexed by the HAM-D28, decreased 24 h following ketamine administration for all participants. The average percent improvement on the HAM-D28 from pre-treatment to 24 h post-treatment across the entire sample was 66.2% (±21.0%). HAM-D28 24 h percent improvement was not correlated with baseline HAM-D28 score (*R* = 0.122, *p* = 0.691), indicating that initial depression severity was not associated with magnitude of clinical improvement. Based on the definition of treatment response typically implemented by clinical trials (an improvement of >50% on the HAM-D28), 10 individuals could be classified as 24 h treatment responders, with an average improvement of 73.9% (±16.9%). The remaining 3 24 h non-responders reported an average HAM-D28 improvement of 40.7% (±9.2%). Rather than utilize a binary treatment response versus non-response classification, this study evaluated clinical response on a continuum in order to better capture the dimensional nature of depression symptoms and symptom improvement.

### Pre-infusion white matter FA is associated with 24 h depression improvement

**Fig. 2:**
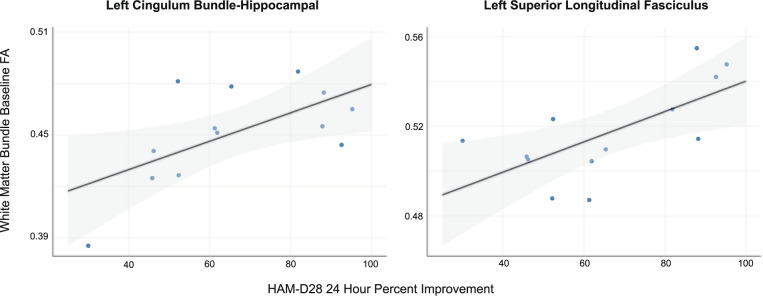
Pre-infusion white matter bundle FA and depression improvement. The relationship between baseline (pre-infusion) white matter FA and clinical improvement at 24 h shown for the left cingulum bundle-hippocampal portion and the left superior longitudinal fasciculus.

Significant positive correlations were found between baseline (pre-infusion) FA and 24 h HAM-D28 percent improvement in the left CB-hippocampal portion (*R*_partial_ = 0.619, *p* = 0.032) and the left SLF (*R*_partial_ = 0.671, *p* = 0.017) (Fig. 2); non-significant positive correlations were observed in 10 of the other 13 bundles examined. There were no significant correlations between baseline FA and baseline HAM-D28 scores in these bundles.

### White matter FA increases from pre- to post-infusion

The average percent change in FA from pre-infusion to post-infusion in the 15 WM bundles is listed in Table 2. Paired samples *t*-tests comparing pre-infusion FA to post-infusion FA in the 15 bundles revealed a significant FA increase for the left ILF (*t* = 2.201, *p* = 0.048), the right ILF (*t* = 3.672, *p* = 0.003), the left SLF (*t* = 2.179, *p* = 0.0499), and the right UF (*t* = 2.326, *p* = 0.038).

**Table 2:** Average pre-infusion to post-infusion percent change in white matter bundle FA.

White matter bundle	Average percent change from pre-infusion to post-infusion (%)	*t*
Right cingulum bundle-hippocampal portion	+1.308	1.745
Right inferior longitudinal fasciculus	+1.247	3.672*
Right uncinate fasciculus	+1.158	2.326*
Left inferior longitudinal fasciculus	+0.901	2.201*
Right inferior fronto-occipital fasciculus	+0.836	2.072
Left superior longitudinal fasciculus	+0.696	2.179*
Left cingulum bundle-cingulate gyrus portion	+0.569	0.756
Left uncinate fasciculus	+0.547	1.299
Right superior longitudinal fasciculus	+0.546	1.502
Left cingulum bundle-hippocampal portion	+0.522	0.573
Left inferior fronto-occipital fasciculus	+0.423	1.415
Corpus callosum-forceps minor	+0.304	0.935
Corpus callosum-forceps major	+0.240	1.218
Right cingulum bundle-cingulate gyrus portion	+0.173	0.295
Fornix	−1.776	−1.525

* indicates significance in the paired samples *t*-test at *p* < 0.05, uncorrected.

### Post-ketamine change in white matter FA is associated with 24 h depression improvement

**Fig. 3:**
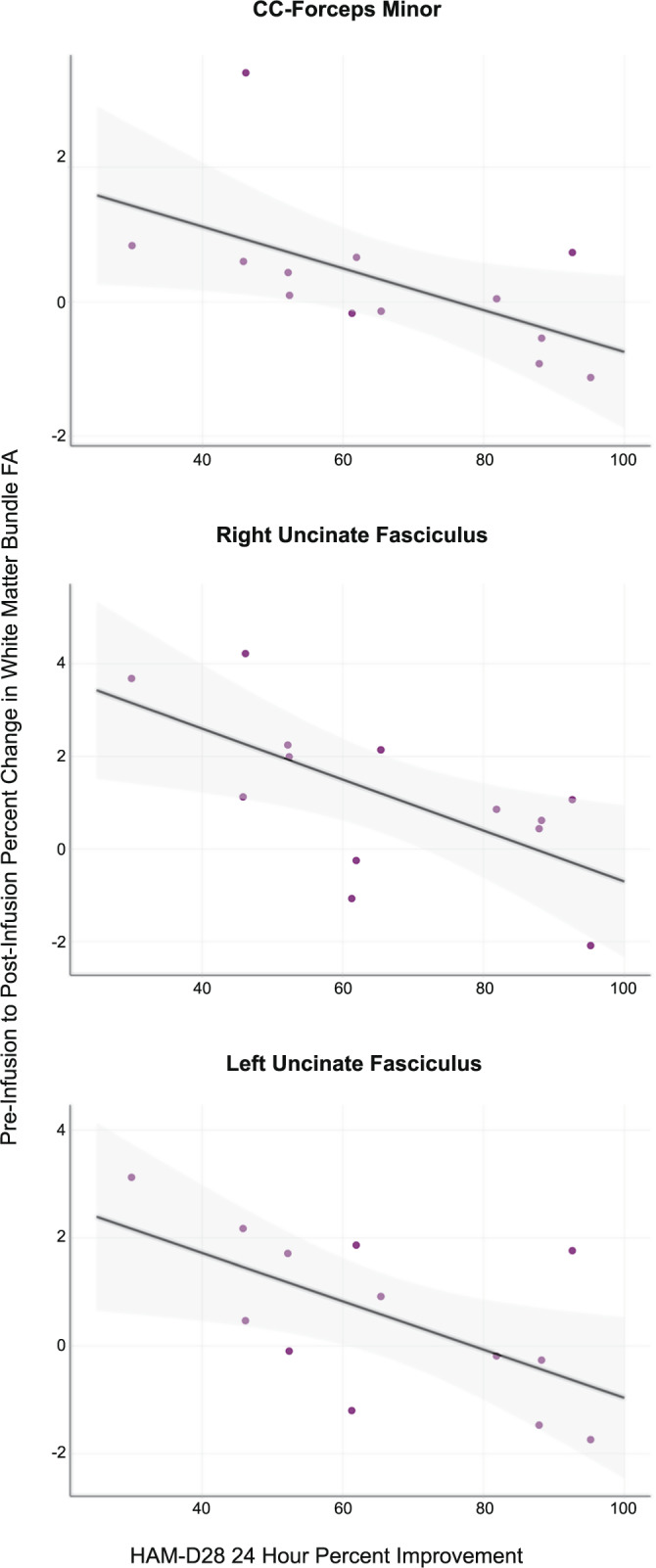
Pre-infusion to post-infusion white matter bundle FA percent change and depression improvement. The relationship between the percent change in white matter FA from pre- to post-infusion and clinical improvement at 24 h shown for the corpus callosum-forceps minor and the left and right uncinate fasciculi.

Significant negative correlations were found between FA percent change and HAM-D28 24 h improvement in the CC-forceps minor (*R*_partial_ = −0.583, *p* = 0.047), the left UF (*R*_partial_ = −0.603, *p* = 0.038), and the right UF (*R*_partial_ = −0.675, *p* = 0.016), indicating that individuals with greater increases in FA reported less improvement in depressive symptoms 24 h following ketamine treatment (Fig. 3). Normality tests revealed, however, that CC-forceps minor—HAMD-D28 association residuals were skewed to the right, likely due to the presence of an identifiable outlier. Upon removal of this outlier residuals normalized and the correlation remained significant (*R*_partial_ = −0.619, *p* = 0.042, *N* = 12). Non-significant negative correlations were additionally observed in 11 of the 12 other bundles examined.

### Sensitivity analyses: assessing head motion and high leverage observations

To ensure that head motion was not significantly impacting the neuroimaging measure under study, we examined associations between head motion and FA. In-scanner head motion was not significantly associated with FA for any of the 15 WM bundles at either time-point (pre-infusion or post-infusion). Further, the paired *t*-test examining differences in head motion between the pre-infusion scan and the post-infusion scan was not significant, indicating that within-subject head motion did not systematically (i.e., directionally) differ between baseline and post-ketamine scans.

One concern with small sample size studies is that a few data points may have an outsized impact on observed associations and corresponding effect sizes (i.e., *R*_partial_). To address this concern, we calculated Cook’s distance for all data points for each significant association reported. We only identified one high leverage data point in the analysis of left CB-hippocampal portion baseline FA and HAM-D28 24 h improvement. No other analyses had high leverage observations.

## Discussion

Results of our pilot study investigating the use of ketamine in TRD support the hypothesis that WM properties may influence or index the antidepressant actions of ketamine. We show that: (1) greater pre-treatment WM FA is associated with larger improvements in depressive symptoms 24 h post-ketamine; (2) ketamine infusion leads to rapid WM diffusivity changes that are evident as early as 4 h post-treatment; and (3) greater post-ketamine increases in WM FA are associated with poorer 24 h symptom reduction. This study thus provides preliminary evidence that ketamine changes WM diffusion profiles, likely as a result of changes in WM microstructure. In addition, these findings suggest that pre-treatment WM structure and the magnitude of treatment-induced changes could potentially be related to ketamine’s efficacy.

Our analysis of dMRI data collected prior to ketamine infusion revealed associations between greater depression symptom improvement at 24 h and higher baseline FA in the left CB-hippocampal portion and the left SLF, two white matter tracts found to be abnormal in dMRI meta-analyses of MDD^25–27^. This finding is a partial replication of Vasavada et al.’s study^16^, in which the authors observed significantly lower WM FA in the cingulum bundle and forceps minor in 24 h ketamine non-responders, as compared to responders. Notably, the present study expands upon the work done by Vasavada et al.^16^ by employing free-water imaging, which corrects tensor measurements for extracellular free-water^18^, as we were able to show that the lower WM FA observed in patients who respond less to treatment is likely attributable to differences in tissue-related processes. This distinction is of particular importance given previous studies demonstrating that decreases in DTI FA can arise from increases in extracellular free-water—even when the microstructure of the tissue itself does not appear to be affected^28^.

Lower FA is often regarded as an in vivo marker of less healthy white matter; however, this certainly need not always be the case^29^. As such, we cannot conclude whether or not the association between lower baseline FA and reduced 24 h response to ketamine treatment arises due to more comprised WM in poorer responders. Rather, a more cautious interpretation of these findings is that FA is indexing a property (or properties) of white matter that is related to ketamine’s antidepressant mechanisms, resulting in a correlation between FA and clinical response to ketamine. Additional preclinical and clinical translational research will need to be undertaken to elucidate what this white matter property is, though WM structural features known to affect FA, including axon diameter, packing density, myelination, and organization, are all possible candidates. Overall, the associations observed between baseline FA and treatment response suggest that characteristics of major limbic and association WM bundles could potentially have an influence on ketamine’s efficacy.

Current evidence supports that ketamine produces its antidepressant effects by increasing the number and the strength of synapses, effectively increasing local synaptic connectivity^8,30,31^. Though largely based on preclinical studies, this may be supported by a functional MRI study demonstrating that ketamine increases functional connectivity of several regions of gray matter with the rest of the brain^32^, a finding that Abdallah et al.^32^ interpreted as reflecting increases in regional synaptic strength. Thus, if ketamine alters depression by inducing increases in local synaptic connectivity that enhance whole-brain functional connectivity, it is plausible that the structure of macroscopic WM connections—which often serve as the structural scaffolding for functional connections—could influence the magnitude of beneficial alterations in brain functioning. At a microscopic level, enhancement of local synaptic connectivity arises due to ketamine’s ability to modulate glutamatergic signaling at NMDA and AMPA receptors^8,30,31,33^. NMDA^34–37^ and AMPA^38,39^ receptors are additionally expressed in WM, and glutamate activation of these receptors can alter WM microstructure^39,40^. It is thus of great interest for future studies to examine the relationship between WM glutamate levels and WM microstructure in TRD within the context of treatment with ketamine. This could be readily accomplished by collecting dMRI along with glutamate chemical exchange saturation transfer (GluCEST) imaging data^41^, which enables white matter-specific quantification of glutamate levels^42^.

A second aim of this pilot was to investigate whether ketamine administration produces rapid changes in WM diffusion profiles. A number of studies have reported that other MDD treatments, including psychotherapy^43^, transcranial magnetic stimulation^44^, and electroconvulsive therapy^45^, alter WM diffusion anisotropy over the course of a few weeks. We were therefore interested in determining whether ketamine, which produces its antidepressant effects within hours instead of weeks, affects FA on this shortened time scale. The results of this study indeed suggest that ketamine may produce alterations in WM FA as early as four hours after treatment. Specifically, within-subject analyses indicated that FA significantly increased from pre- to post-infusion in the majority of individuals in the right ILF (increased in 9/13 subjects), the left ILF (11/13 subjects), the right UF (10/13 subjects), and the left SLF (10/13 subjects). Although these were the only bundles to show a significant within-subject increase in FA following ketamine administration, FA increased, on average, from pre- to post-infusion in 14 of the 15 WM bundles examined. We posit that the effect of ketamine on WM in the brain is likely global (rather than bundle-specific), but that our sample size (considered in conjunction with intrinsic variability in neurobiological responses to psychotropics) may be too small to detect statistically significant FA changes across all bundles. Finally, although the magnitude of these FA changes may seem small (an average increase of ~1%), it is in line with previously observed ketamine-induced changes in neuroimaging measures, e.g., with a 1% increase in the resting BOLD signal observed in individuals receiving a ketamine infusion in the scanner^46^.

Relevant to contextualizing these FA increases post-ketamine, it has been suggested that diffusion measures may undergo time of day-dependent fluctuations. Studies exploring this possibility have reported either no significant changes in FA between the morning and the afternoon^47^, or a decrease in FA from the morning to the evening^48^, i.e., a diurnal change in FA opposite in direction to the increase in bundle FA observed following ketamine administration. Critically, time of day related changes in DTI metrics appear to be explained by diurnal fluctuations in extracellular CSF and free-water^47^. As we have removed the free-water signal from the calculation of FA, time of day changes in dMRI measures are unlikely to explain our results of ketamine-induced changes in WM FA.

As previously discussed, WM FA is affected by axon density, myelination, and organization, and changes in anisotropy are often interpreted as changes in these axonal features^29,49^. In the present study, however, the 4 h time frame is too short for substantial changes in myelination or axonal organization to occur, thus the FA changes observed following ketamine are likely the result of other neurobiological changes that can occur rapidly in WM. While the nature of these changes cannot be resolved from this study, we propose that astrocyte plasticity may contribute to the observed FA changes^49^, especially given that astrocytic processes can undergo fast structural changes, extending and retracting within minutes^50^.

In WM, the diffusion signal is influenced not only by axons and myelin, but also by other cellular components such as astrocytes. In a rodent study designed to elucidate biological contributors to the diffusion signal, Walhovd et al.^51^ found that in WM, astrocytes make up roughly the same proportion (~48%) of the volume of a voxel as myelin. The authors concluded that astrocytes must account for a significant portion of the diffusion signal acquired from a 2 mm^3^ WM voxel in the human brain. Furthermore, they posited that changes in the number, organization, or thickness of astrocytic processes could impact diffusion measurements—an idea corroborated in a rodent study using histology and DTI^52^. More specifically, increases in the number, length, or thickness of WM (“fibrous”) astrocytic processes should lead to increases in FA, as these processes are aligned directionally with myelinated axons. Accordingly, we propose that the FA increases observed following ketamine infusion in this study could potentially reflect WM astrocyte plasticity. We emphasize that this explanation is speculative and primarily intended to be hypothesis-generating, as dMRI characterization of water diffusion is not cell-type specific. Still, this explanation is supported by preclinical literature demonstrating that ketamine administration increases the number and length of astrocytic processes and expression of glial fibrillary acidic protein (GFAP; an intermediate filament highly expressed in astrocytes)^9,10^. If astrocytic morphological changes do indeed underlie some of the observed post-treatment increases in FA, our results seem to suggest that larger increases in fibrous astrocytic processes are associated with less symptom improvement following ketamine. The nature of this relationship requires further investigation in additional neuroimaging and animal model studies of ketamine. Finally, while preclinical work supports that the dMRI changes observed following ketamine potentially arise due to astrocytic microstructural changes, it is additionally possible that decreases in cell membrane water permeability could contribute to post-treatment increases in FA^29,53^. Future endeavors should aim to differentiate between these two mechanisms by harnessing diffusion-weighted magnetic resonance spectroscopy of myo-inositol^54^ and permeability diffusivity imaging^13,55^, methods which provide in vivo measures of astrocyte morphology^54,56^ and membrane permeability, respectively.

### Limitations

Due to the preliminary nature of this study and the ambitious study design, the size of the sample is small, which can lead to inflated effect sizes. Further, as this was an exploratory, whole-brain analysis of ketamine and WM structure conducted in a small sample, we did not correct *p-*values for multiple comparisons across all 15 bundles. Replication in a larger, independent sample is thus needed in order to validate or refute these preliminary findings. The dMRI measure examined, FW-corrected FA, is a sensitive but non-specific measure. Thus, we cannot experimentally determine the precise neurobiological changes that underlie a change in FA. Furthermore, given that this study only included a follow-up scan on the day of the infusion, we cannot determine if the FA changes observed at 4 h persist over time, or whether they instead represent a transitory change following ketamine. The interpretation of the ketamine-induced changes in FA are potentially limited by the lack of a control group of individuals who did not receive an infusion of ketamine. However, previous within-subject, same day longitudinal studies of healthy individuals demonstrate that the increases in FA observed following ketamine are not consistent with (and in fact opposite in direction to) previously observed diurnal changes in FA^48^. Last, the population we studied was heterogeneous in terms of age, sex, clinical symptoms, and the type of antidepressant trial(s) failed. However, given the limited sample size, we could not examine if and how these factors affect relationships between WM and treatment outcome.

## Conclusion

This pilot study employed diffusion imaging to investigate relationships between WM and ketamine treatment for individuals with TRD, toward the goal of improving our understanding of the antidepressant mechanisms of ketamine. The present analyses corroborate previous findings that the structure of the brain’s WM network may affect an individual’s response to ketamine treatment, while further providing initial evidence that ketamine can rapidly alter WM diffusion properties as measured with dMRI. Additional investigations into the effects of ketamine on WM and relationships to clinical outcomes appear warranted. Such investigations will continue to provide new insight into rapid-acting treatments for MDD.

## Data Availability

Analytic code is available at github.com/valeriejill/ketamine_TRD_dMRI.
